# Dietary chitosan promotes the growth, biochemical composition, gut microbiota, hematological parameters and internal organ morphology of juvenile *Barbonymus gonionotus*

**DOI:** 10.1371/journal.pone.0260192

**Published:** 2021-11-18

**Authors:** Mohammad Abdus Salam, Md. Ashikur Rahman, Sulav Indra Paul, Fatama Islam, Avishek Kanti Barman, Zinia Rahman, Dinesh Chandra Shaha, Md. Mahbubur Rahman, Tofazzal Islam

**Affiliations:** 1 Faculty of Fisheries, Department of Genetics & Fish Breeding, Bangabandhu Sheikh Mujibur Rahman Agricultural University, Gazipur, Bangladesh; 2 Institute of Biotechnology and Genetic Engineering, Bangabandhu Sheikh Mujibur Rahman Agricultural University, Gazipur, Bangladesh; 3 Faculty of Fisheries, Department of Fisheries Management, Bangabandhu Sheikh Mujibur Rahman Agricultural University, Gazipur, Bangladesh; Kafrelsheikh University, EGYPT

## Abstract

In this study, we determined the effects of dietary chitosan on the growth, biochemical composition, gut microbiota, and hematological and histological parameters of juvenile *Barbonymus gonionotus*. Three test diets containing three different concentrations (1, 2, and 3 g kg^-1^ feed) of dietary chitosan were formulated. A basal diet without dietary chitosan was considered a control, and the fish were reared for 60 days. Comparing the effects of the dietary chitosan-containing diets with those of the control diet, we found that dietary chitosan significantly improved the muscle growth, nutrient and mineral contents, hematological parameters, lactic acid bacterium abundance, and digestive enzyme activities of *B*. *gonionotus*. Moreover, dietary chitosan significantly inhibited the growth of pathogenic bacteria in fish. Interestingly, an increase in the dietary chitosan level significantly enhanced the protein contents of the muscles and inversely significantly decreased the lipid contents compared to those with the basal diet. Quantitative study revealed that dietary chitosan significantly enhanced the length of intestinal villi, and qualitative study showed that dietary chitosan considerably reduced the fat content in the liver and improved the morphology of the kidney compared to those with the basal diet. Taken together, our results suggest that the application of dietary chitosan at a dose of 1 g kg^-1^ feed produced the highest benefit to treated *B*. *gonionotus*, indicating its potential for safe use in aquaculture.

## Introduction

Aquaculture plays many significant roles in sharply enhancing fish production to meet the growing protein requirement demands of the ever-increasing population of the world. Importantly, aquaculture has expanded in more professional ways, and intensive aquaculture systems are available in which artificial feeding is a major component. Recently, fishery production has plateaued, whereas the demand for fish production is multiplying. In this context, aquaculture has come to represent one of the main sources of fish for human nutrition. Interestingly, aquaculture production has increased very quickly in the last few decades. It was the source of 51.4 million tons of food fish in 2016, which was 64.2% of the world’s farmed food production, compared to 57.9% in 2000 [1]. *Barbonymus gonionotus* is a minor carp and it is known as silver barb in aquaculture. It is also an exotic fish of Bangladesh belonging to the Cyprinidae family. This fish species can endure the adverse environmental condition [2,3] and it has high productivity [3] and good taste, which make it a popular culturable species for the enhancement of annual fish production in Bangladesh. It has the highest protein content among freshwater fishes [4]. To continue aquaculture production, it is necessary to develop nontraditional fish diets that contain natural and inexpensive ingredients and to enhance the physiological and biological functions of farmed fishes. Bacterial diseases are a common threat to the aquaculture industry [5]. Therefore, fish farmers are compelled to use antibiotics to mitigate bacterial diseases in aquafarms. However, the use of antibiotics has been met with increasing opposition because of their negative long-term effects on the environment and potential harm to human consumers [6]. Recently, aquaculture feeds have been a major focus to enhance fish production safely. Unbalanced formulated feeds may cause serious problems to human health. In this regard, supplementation with the biopolymer chitosan could play a significant role in increasing fish production and improving the quality of feeds in aquaculture in a safe way.

Chitosan, β (1–4)-linked 2-acetamido-2-deoxy-β-D-glucose (N-acetyl glucosamine), is a broadly used natural compound [7]. This is an essential biopolymer, utilized in various fields like agriculture and the industrial purposes [8]. It can be applied as a feed additive and it advances growth, resistant capacity, intestinal microbial microorganism restraint, and cholesterol level decrease of terrestrial animals [9,10]. Prebiotic dietary chitosan also exerts beneficial effects on treated fish in aquaculture. Many scientists have demonstrated that the application of probiotics promotes growth, the immune system, and hematological parameters and suppresses fish diseases [11–14]. However, little information is available on the effect of dietary chitosan from finfish scales, crustacean shells, and the fungal cell wall on the growth promotion and health improvement of fishes.

Dietary chitosan-incorporated diets help to improve the growth performance [15–18] and reduce the mortality of fishes [15,17]. The application of dietary chitosan with formulated feeds also enhances protein and decreases lipid and moisture contents in fishes [19,20]. Dietary chitosan-treated formulated feed also affects lipid metabolism in fishes [21] and mineral contents in humans [22]. Interestingly, dietary chitosan-incorporated diets also affect the intestinal histology [16,19,23] and blood parameters, gut enzyme activity, and innate immunity [18,19,23–26] of different fishes at different dosages. However, data on the effects of dietary chitosan on the growth performance, biochemical composition, hematological parameters, gut microbiota, and internal organ morphology of juvenile *B*. *gonionotus* are lacking. The objectives of the present study were to determine the effects of formulations with varying levels of dietary chitosan on i) growth, biochemical composition, and hematological parameters; ii) gut microbiota status, digestive enzymes and pathogenic bacteria; and iii) the intestine, liver, and kidney histology of juvenile *B*. *gonionotus* fish.

## Materials and methods

### Ethics statement

The use of animals was kept to the absolute minimum required to achieve statistical significance for validation purposes. All procedures were conducted in accordance with the United Kingdom Animal (Scientific Procedures) Act 1986; approved by the Ethical Review Committee (ERC) of the Institute of Biotechnology and Genetic Engineering (IBGE), Bangabandhu Sheikh Mujibur Rahman Agricultural University (BSMRAU), Gazipur-1706, Bangladesh; and conducted under approval no. BSMRAU/IBGE/003. This study was performed in compliance with the ARRIVE guidelines 2.0 [27]. Declarations are available as S1 File.

### Euthanasia methods

Clove oil (Sigma C8392) was utilized in anesthetization and euthanization of the fish. Clove oil preparation was ready as per Fernandes et al. [28]. Briefly, pure clove oil was first dissolved in ethyl alcohol in a 1:9 ratio (clove oil:ethyl alcohol). This solution was then weakened in water to get convergences of 0.05 milliliter (mL) (50 milligram (mg) and 0.20 mL (200 mg) of clove oil per 500 mL of water. For histological and intestinal microflora considers, fish were euthanized by utilizing 0.20 mL of clove oil per 500 mL of water, and passing was affirmed by the annihilation of the cerebrum [28,29].

### Collection of experimental fish

A total of 360 (average 20.9 ± 0.56 g) juvenile experimental fish (*B*. *gonionotus*) were collected from the private fish farm Bismillah Fish Hatchery, Trishal, Mymensingh, Bangladesh, and transported to the wet laboratory of Genetics and Fish Breeding, BSMRAU, Gazipur, Bangladesh, with the provision of continuous aeration. Handling, maintenance, and release of all experimental fish were performed following the Canadian Council on Animal Care guidelines [29]. Briefly, the fish were stocked in circular plastic tanks (500 L) with aerators and acclimatized for 15 days in the wet laboratory according to Mohapatra et al. [30]. Water changing was performed every 3 days, and uneaten feed was collected. Water quality parameters such as pH, dissolved oxygen, and temperature were routinely measured to maintain the health of the fish. During acclimatization, the fish were taken care of according to Salam et al. [31] until the trial began.

### Experimental design, conditions, and feeding trial

To assess the effect of dietary chitosan in formulated feed, twelve plastic tanks (500 L) were divided into four treatments: control, T1, T2, and T3. Each treatment had three replicates. The control treatment lacked supplementation with chitosan (0 g kg^-1^ feed) in the formulated feed. The T1, T2, and T3 treatments included supplementation with three concentrations of chitosan at doses of 1 g kg^-1^, 2 g kg^-1^, and 3 g kg^-1^, respectively. A total of 360 uniformly sized juvenile fish were randomly distributed among the four treatments, and the stocking density was maintained at 30 fishtank^-1^ following a completely randomized design. The fish were acclimatized to commercial feed for 15 days. After the acclimatization period, the control treatment fish were fed without dietary chitosan, and the T1, T2, and T3 fish were fed with the addition of three concentrations of chitosan at doses of 1 g kg^-1^, 2 g kg^-1^, and 3 g kg^-1^, respectively, for an experimental period of 60 days.

The fish were hand-fed at 6% of the total biomass, twice daily at 0900 h and 1900 h, for 60 days. Feed adjustments were made for each tank every 15 days after sampling. The dough was prepared every 7 days interval. The uneaten feed was collected during water exchange. During the experiment, uneaten feed was collected regularly to calculate the exact feed intake of the experimental fish. Treatment-wise feed consumption percentages were as follows: control (80%), T1 (87%), T2 (85%) and T3 (83%). The nutrient compositions of exact feed intake by the fish are presented in S1 Table.

### Chitosan collection

Laboratory-grade chitosan biopolymer (poly β-1, 4-D-glucosamine) available in powder form was purchased from RESEARCH-LAB FINE CHEM INDUSTRIES, Mumbai 400–002, India (cat. No. 2095A). The chitin collected from shrimp shells was prepared by the alkaline deacetylation. The properties of this biopolymer are degree of deacetylation > 70%, viscosity (1% chitosan in 1% acetic acid solution at 28°C) > 100 Click-Per-Second (CPS), ash < 1%, insoluble <1%, moisture < 10% and pH 6.5 to 9.

### Preparation of control and dietary chitosan-treated feeds

The chitosan-supplemented diets were prepared according to Zaki et al. [15]. For the preparation of formulated feed, the feed ingredients were locally collected from Joydebpur Bazar, Gazipur. Feed was formulated using fish meal, soybean meal, maize flour, mustard oil cake, wheat flour, molasses, and vitamin-mineral premix. Isonitrogenous (37% crude protein) and isolipidic (8% lipid) diets were formulated (Table 1). Dietary chitosan was not added to the control diets. Approximately 37% protein was maintained in the diet through the formulation of feed following Pearson’s square method. Required amounts of ingredients were weighed using a digital balance, and all the feed ingredients were thoroughly mixed manually. Vitamin-mineral premixes and chitosan were separately mixed and gradually added to the dry mixture. Then, those ingredient mixtures with vitamin-mineral premixes were ground through a blender. After blending, all of the feed ingredients were mixed. Then, feeds were prepared in pellet (size 4 mm) form using an aluminum wire sieve, dried at room temperature, packed in plastic small plastic bags, and stored at 4°C to maintain microbial viability, according to Allameh et al. [32]. Feeds were repeatedly prepared every 7 days during the experiment [33]. The proximate composition of formulated feed (moisture, protein, ash, lipid, and fiber) was analyzed for the chitosan-treated feed as well as for the control feed (Table 2) [34].

**Table 1:** Composition of experimental feed addition with the graded level of dietary chitosan for the rearing of juvenile *B*. *gonionotus*.

Ingredients	Inclusion level (%)			
	Control	T1	T2	T3
Fish meal^1^	28.90	28.80	28.7	28.60
Soybean meal^2^	28.00	28.00	28.00	28.00
Mustard oil cake^3^	15.00	15.00	15.00	15.00
Maize flour	12.10	12.10	12.10	12.10
Wheat flour	10.00	10.00	10.00	10.00
Molasses	5.00	5.00	5.00	5.00
Vitamin mineral premix^4^	1.00	1.00	1.00	1.00
Chitosan (g kg^-1^ feed)^5^	0	0.10	0.20	0.30

^1^ Locally purchased, crude protein 70%, crude lipid 9%.

^2^ Mega Feed Limited, Bangladesh, crude protein 49%, crude lipid 20%.

^3^ Locally purchased, crude protein 40%, crude lipid 20%.

^4^ Vitamin premix (mg kg^-1^ diet): Thiamin, 25; riboflavin 20; pyridoxine 21; cyanocobalamin, 0.03; folic acid 5; calcium pantothenate, 45; inositol, 100; niacin 100; biotin 0.1; starch, 3400; ascorbic acid, 100; vitamin A, 100; vitamin D, 20; vitamin E, 50; vitamin K, 12.

^5^ Chitosan was purchased from Research-lab Fine Chem Industries, Mumbai 400–002, India.

**Table 2:** Estimation of the proximate composition of control feeds and individual graded levels of dietary chitosan-treated feeds (dry matter basis) for rearing *B*. *gonionotus*.

Treatment	Dry matter (%)	Lipid (%)	Protein (%)	Ash (%)	Crude fiber (%)	Carbohydrate/NFE (%)
Control	88.12	10.87	37.27	14.21	6.13	31.52
T1	88.75	9.99	37.11	14.37	6.09	32.44
T2	88.74	10.1	37.27	14.45	5.99	32.19
T3	88.23	10.09	37.4	14.05	6.30	32.16

Here, NFE = nitrogen free extract.

### Water quality parameters

The temperature, dissolved oxygen (DO), and pH of the water were recorded every day. Temperature and DO were estimated by a computerized thermometer and DO meter (LUTRON PDO-519, TAIWAN), individually. The pH was estimated by a versatile advanced pH meter (EZODO, pH 5011). Water quality parameters in the range of pH 7.3–8.4, DO 5.3–5.6 mgL^-1^, and temperature 26.5–30°C were maintained throughout the experimental period [35], and those ranges were within the optimum water quality parameters for juvenile *B*. *gonionotus* fish [36,37].

### Measurement of growth parameters and feed efficiency of juvenile *B*. *gonionotus*

To survey the growth status, the fish samples were gathered from a business aquafarm in Trishal, Mymensingh, Bangladesh. A total of 90 fish (each replication included 30 fish) were taken for each treatment. The growth of juvenile *B*. *gonionotus* was evaluated in terms of body weight gain (%BWG), specific growth rate (SGR, % day^-1^), and feed efficiency, which was evaluated by calculating the food conversion ratio (FCR) value. Sampling was accomplished every fortnightly to check the growth status and adjust the amount of feed for the new fish.

Weight gain in grams = mean final weight in grams—mean initial weight in grams.

BWG (%) = (final weight−initial weight)/initial weight ×100

$$SGR\;(\%/day)=\frac{lnW_{2}-lnW_{1}}{T_{2}–T_{1}}\times 100$$

Where lnW_1_ = the initial live body weight (g) at time T_1_ (day)

lnW_2_ = the final live body weight (g) at time T_2_ (day)

FCR: feed conversion ratio = (feed intake (g) wet weight gain (g)^-1^).

### Chemical analysis of carcass composition of *B*. *gonionotus*

Toward the finish of the growth preliminary exploratory period (60 days), the fish were gathered for the proximate examination. Nine fish from each treatment were anesthetized with clove oil and gradually sacrificed [28]. The fresh fish were weighed by an electric equilibrium. Then, at that point, the fish were dried for the time being utilizing a stove at 105°C (MC2846SL, LG Company, India) for 12 h, and the dried fish weight was estimated once more. Then, at that point, the moisture content was controlled by deducting the weight of dried fish from the weight of new fish. Then, the dried fish samples were ground using an electrical blender. To determine crude protein, the nitrogen content of the fish carcass was measured according to the modified Kjeldahl method following sulfuric acid (H_2_SO_4_)-salicylic acid digestion, distillation, and titration [38]. The fat content in the carcass was determined by Soxhlet extraction with diethyl ether [39]. The ash content was dictated by incineration of the carcass at 550°C for 12 h in a muffle heater (Hayashi Denko Co., Ltd., Japan) [39].

### Investigation of mineral contents in the muscles of *B*. *gonionotus*

At the end of the growth trial experimental period (60 days), the fish were collected for the analysis of mineral contents in the muscles of fish. The trial fish were sympathetically killed by utilizing clove oil (0.20 mL per 500 mL of water) [28], and demise was affirmed by the obliteration of the cerebrum [29]. Nine randomly selected fish from each treatment were anesthetized with clove oil and gradually sacrificed [28]. The mineral contents, such as those of potassium (K), sodium (Na), calcium (Ca), magnesium (Mg), zinc (Zn), iron (Fe), and manganese (Mn), of the fish muscles were analyzed according to Piper [40]. In brief, the fish muscles were collected by removing the head, fins, scales, and intestine. Then, at that point, the muscles were dried utilizing a stove at 105°C (MC2846SL, LG Company, India) for 12 h, and the dried muscles were ground utilizing a blender. The powdered samples were digested in a boiling nitric acid and perchloric acid mixture (5:1) by following standard methods. After fitting dilution, the mineral contents, like those of K, Na, Ca, Mg, Zn, Fe, and Mn, of fish muscles were assessed utilizing a nuclear assimilation spectrometer (170–30, sequential 6268–001, Hitachi, Japan). Aligned principles for mineral assessment were ready from financially accessible guidelines (Buck Scientific 1-800-562-5566, BS-AQ-PB, single component AA standard, USA). The assessments of K, Na, Ca, Mg, Zn, Fe, and Mn substance were performed utilizing a hollow cathode light (Hitachi, Japan).

### Assessment of the effects of chitosan on the gut microbiota of juvenile *B*. *gonionotus*

The gut microbial flora of fish was analyzed according to Hoseinifar et al. [41], with some modifications. The analysis was accomplished at the start of the feeding trial by random sampling of 24 fish. The exploratory fish were sympathetically killed by a sedative excess utilizing clove oil (0.20 mL per 500 mL of water) [28]. At the end of the feeding trial for growth performance, 18 randomly sampled fish from each treatment (6 fish from each replication) were examined to assess the effect of dietary chitosan on the gut microbiota.

### Evaluation of *in vitro* antimicrobial properties of dietary chitosan against fish pathogens

To assess the *in vitro* antimicrobial properties of chitosan, four fish pathogenic bacterial strains were selected. One gram of dietary chitosan was dissolved in 5 ml of dimethyl sulfoxide (DMSO), and nutrient broth was prepared for inoculation. This experiment was designed into three treatments *viz*. control (without DMSO), DMSO-supplemented broth (T4), and a mixture of DMSO and chitosan in broth (T5). Each treatment included three replicates. Then, the pathogenic laboratory strains were inoculated into the nutrient broth. DMSO (0 μl), DMSO (100 μl), and a mixture of DMSO and chitosan (100 μl) were added to the broth. Then, all broth cultures were incubated at 28°C for 24 h with 100 rpm rotation in a shaker incubator. After that, 100 μl of each of the broth cultures was spread on duplicate nutrient agar plates and incubated at 28°C in an incubator. After incubation, colony-forming units (CFU) were calculated following Mohapatra et al. [30].

### Assessment of the effect of chitosan on the intestinal digestive enzyme activities of *B*. *gonionotus*

The effects of dietary chitosan on intestinal digestive enzyme activities were assessed through enzyme assays as previously described by Gao et al. [42], with some modifications. At the end of a 60-day trial, the fish of each tank were bulk-weighed after starving for 24 h. Nine fish of each treatment were randomly sampled to assay the intestinal digestive enzyme activity of *B*. *gonionotus*. Nine fish of every treatment were haphazardly inspected to measure the intestinal digested enzyme activity of *B*. *gonionotus*. Fish were forfeited by an excess of clove oil, and the outside of each fish was sanitized utilizing 70% ethanol. The peritoneal cavity was opened aseptically with a sterile surgical tool. The digestive tract between the pyloric caeca and roughly 1 cm foremost to the anus of the fish was extracted, and the dung with bodily fluid were peeled off with sterile forceps.

The intestine of 9 fish from each treatment was removed and frozen in liquid nitrogen and then stored at -80°C until subsequent analysis. The intestinal samples were homogenized in 10 vol (v/w) of ice-cold phosphate-buffered saline (PBS) and centrifuged at 3000 ×g for 20 min at 4°C. The supernatant was conserved and used to determine amylase activity, protease activity, and lipase activity using enzyme-linked immunosorbent assay (ELISA) kits (Thermo Fisher Scientific, USA).

### Histological analyses of the intestine, liver, and kidney of the dietary chitosan-treated fish

At the end of the growth trial experimental period (60 days), fish were collected for histological study. The test fish were sympathetically killed by utilizing clove oil (0.20 mL per 500 mL of water) [28,43], and demise was affirmed by the obliteration of the cerebrum [29]. Nine fish from every treatment were anesthetized with clove oil and bit by bit forfeited to gather the digestive tract, kidney, and liver for histological investigation [28]. After sacrificing the fish, the whole liver, kidney, and part of the intestine from each fish were dissected carefully, cut to separate from each other, and stored in Bouin’s solution for 24 h. Then, at that point, these samples were got dried out in rising grades of ethanol and cleared in xylene. The decent tissues were inserted in histoparaffin (Paraplast Plus; Sigma-Aldrich), and segments (7 μm) were cut utilizing a microtome (CUT-5602, Germany). Then, at that point, sections of intestinal villi and liver were chosen and stained with Delafield’s hematoxylin-eosin for perception under a light magnifying instrument (DM 100; Leica, Wetzlar, Germany). Ten slides were prepared from the intestine of each fish through the histological method. Each slide contained ten intestinal tissue sections. Then, at that point, the slides were seen under a trinocular magnifying lens. The pictures were caught utilizing a computerized camera (DFC 290, Leica), and the villus length of the digestive system was estimated utilizing AmScope programming (adaptation 3.7; Carl Zeiss Primo Star, Germany).

### Measurement of hematological parameters

Blood samples were collected from the experimental fish according to the Canadian Council on Animal Care [29,43]. At the end of the growth trial experimental period (60 days), a total of 90 fish from each treatment were anesthetized with clove oil (0.05 mL per 500 mL of water) for hematological analysis [28]. Blood was collected from fish using a 3 cc syringe containing 10% blood anticoagulant ethylenediamine tetraacetic acid (EDTA) inserted into the caudal peduncle region to draw out blood. The blood was moved to a test tube covered with EDTA and put away at—30°C until use. Red blood cells (RBCs) and white blood cells (WBCs) were checked utilizing a further developed Neubauer hemocytometer (MarienFeld Company, Germany) under a light magnifying instrument (DM 100; Leica, Wetzlar, Germany) as indicated by Shah and Altindağ [44]. To quantify hemoglobin content, new blood was gathered from the fish of every treatment and poured in the edge of a strip of hemoglobin meter before coagulation. The glucose level of blood samples was measured through a glucose meter. To measure packed cell volume (PCV) (%), blood was collected in a capillary tube to the marked level and sealed with gum. The capillary tube was placed in the rotor of a hematocrit machine at a sealed point in the outward direction, and the machine was allowed to run for 5 min at 15,000 rpm. The length of the blood cells was measured by a hematocrit measuring scale, and the recorded value was multiplied by the concentration of blood. Packed cell volume (PCV) (%) = hematocrit value **×** blood concentration **×** 100.

### Statistical analysis

All data (body weight gain, weight gain, SGR, %BWG, FCR, and moisture, protein, lipid, and ash and mineral contents of the carcass, digestive enzyme activitity, blood parameters, and the gut microbiota) were collected during the study period and statistically analyzed using one-way analysis of variance (ANOVA) to test the significance of results (P < 0.05) between means, and the mean values were separated by least significant difference (LSD) post hoc statistic. The standard deviation (±SD) was calculated to identify the range of means. Fish carcass mineral contents were statistically analyzed using one-way analysis of variance (ANOVA), linear, and quadric trends to test the significance of the experimental results (P < 0.05) between means [45]. All statistical analyses were performed with the aid of the computer software Statistix version 10.0. Power analysis was performed to check the statistical validity of the sample size. The typical power analysis for ANOVA was performed using G*Power version 3.0.10 according to Faul et al. [46].

## Results

### Effects of dietary chitosan on the growth and feed efficiency of juvenile *B*. *gonionotus*

In the case of chitosan application, the weight gains of fish were 26.11 ± 1.40, 39.40 ± 1.47, 34.37± 1.24, and 30.17 ± 0.60 g in the control, T1, T2, and T3 groups, respectively, after the end of 60 days (Table 3). Significantly, the highest body weight gain was found in fish fed feed supplemented with dietary chitosan at a dose of 1 g kg^-1^ at days 15, 30, 45, and 60 after the treatment (Table 3). Strangely, a diminishing pattern of body weight gain was found in the treatment of fish with higher portions of dietary chitosan than 1 g kg^-1^ feed (Table 3). The statistical analyses also showed a similar trend among the treatments; the highest weight gain was found in fish fed feed supplemented with dietary chitosan at the T1 dose (1 g kg^-1^ feed) after 60 days (Table 3**)**. Surprisingly, a decreasing trend of body weight gain was found in the treatment of fish with higher doses of dietary chitosan than 1 g kg^-1^ feed (Table 3). A similar trend for the effects of dietary chitosan was also found for the percent body weight gain (%BWG) and specific growth rate (SGR%/day) of fish (Table 3). The feed conversion ratios (FCRs) in the control, T1, T2, and T3 groups were 1.48 ± 0.022, 1.2 ± 0.029, 1.3 ± 0.020, and 1.38 ± 0.008, respectively, after 60 days (Table 3). A reverse trend of results was obtained in the case of the FCR (Table 3). The most elevated FCR was recorded in the fish treated with no dietary chitosan. On the other hand, the FCR was significantly increased with increasing doses of dietary chitosan (Table 3).

**Table 3:** Growth parameters of fish fed in indigenous feed ingredients formulated feeds with graded levels of dietary chitosan feeding trial for 60 days.

**Period in day and BWG**	**Control**	**T1**	**T2**	**T3**
Initial BWG (g)	19.87 ± 0.19^a^	20.16 ± 0.60^a^	19.82 ± 0.24^a^	20.08 ± 0.27^a^
15-day BWG (g)	25.56 ± 0.45^c^	30.11 ± 0.42^a^	28.16 ± 0.92^b^	27.53 ± 1.43^b^
30-day BWG (g)	31.29 ± 0.33^d^	40.12 ± 0.35^a^	36.62 ± 0.89^b^	34.82 ± 0.95^c^
45-day BWG (g)	38.24 ± 0.90^d^	50.42 ± 0.49^a^	45.29 ± 0.50^b^	42.64 ± 0.93^c^
60-day BWG (g)	45.98 ± 1.20^d^	59.56 ± 0.61^a^	54.21 ± 0.80^b^	50.26 ± 0.71^c^
**Growth parameters**	**Control**	**T1**	**T2**	**T3**
Initial wt. gain(g)	19.87±0.19^a^	20.16±0.60^a^	19.82±0.24^a^	20.09±0.27^a^
Final wt. gain (g)	45.98±1.20^d^	59.56±0.61^a^	54.20±0.80^b^	50.27±0.71^c^
Weight gain (g)	26.11±1.40^d^	39.4±1.47^a^	34.37±1.24^b^	30.17±0.61^c^
% BWG	131.43±6.86^d^	195.82±14.15^a^	173.54±8.7^b^	150.22±2.19^c^
SGR (%/day)	1.39±0.04^d^	1.80±0.08^a^	1.68±0.53^b^	1.53±0.014^c^
FCR	1.45±0.022^a^	1.20±0.029^d^	1.30±0.02^c^	1.38±0.006^b^

Here, Control = 0 g kg^-1^ feed; T1 = 1 g kg^-1^ feed; T2 = 2 g kg^-1^ feed and T3 = 3 g kg^-1^ feed. One-way ANOVA was performed to analyze the data of three replicate experiments, and the data in the columns varied significantly according to the least significant difference (LSD) at P < 0.05 (Statistix 10). Different letter bars indicate significant variations in body weight gain (BWG), initial weight gain, final weight gain, weight gain, % body weight gain (% BWG), specific growth rate (SGR), and feed conversion ratio (FCR) of the fish in different dietary chitosan groups at P < 0.05 (Statistix 10). Error bar = ±SD; n = 90.

### Influence of dietary chitosan on the length of intestinal villi of juvenile *B*. *gonionotus*

**Fig 1:**
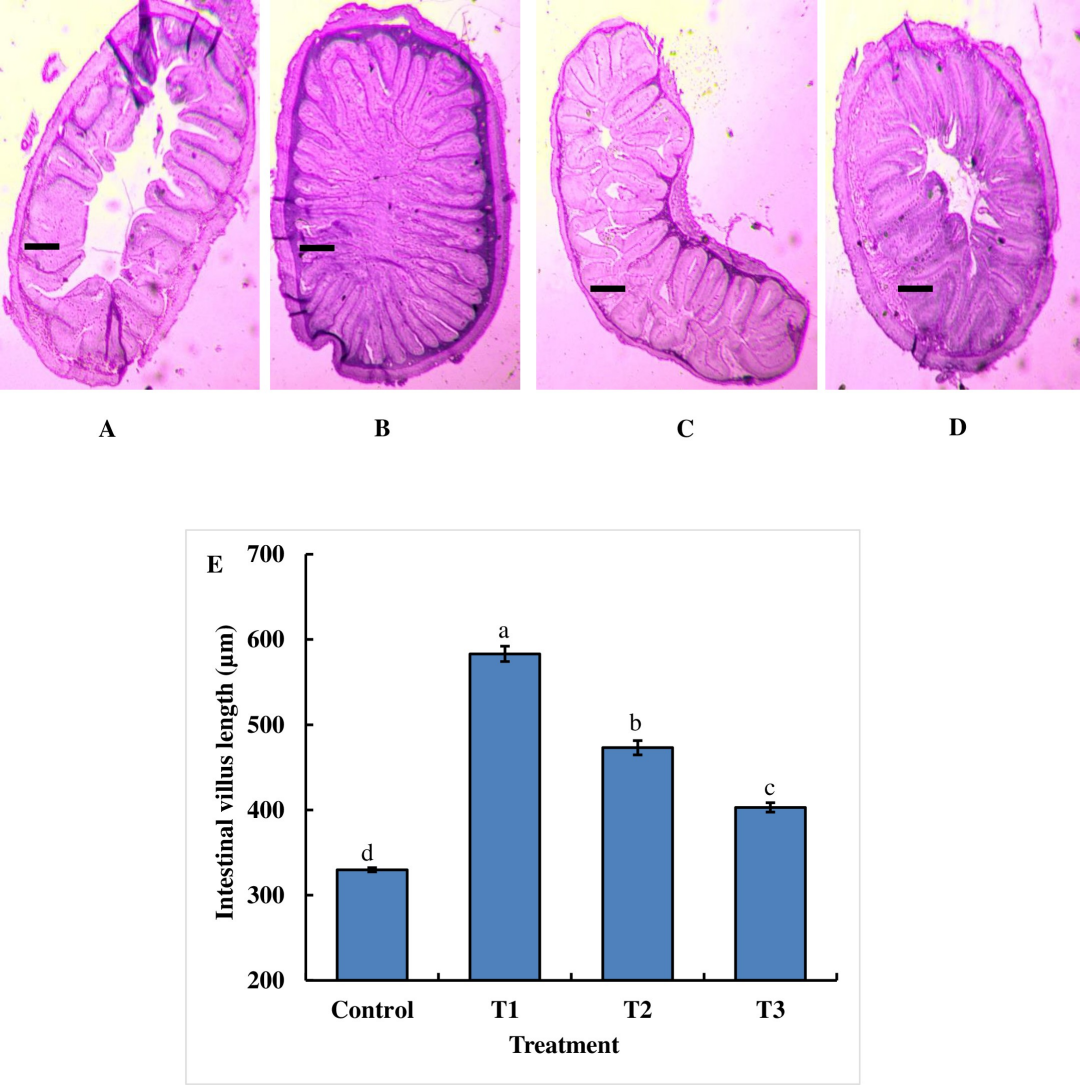
Histological analysis of the effects of dietary chitosan on villus length in the intestine of *B*. *gonionotus* (A-E). Fish were fed a (A) the control diet (0 g kg^-1^ feed), (B) T1 (1 g kg^-1^ feed), (C) T2 (2 g kg^-1^ feed) or (D) T3 (3 g kg^-1^ feed). (E) Dietary chitosan-treated intestinal villus length compared to that with the control is shown (n = 9). Different letter bars indicate significant variations in the intestinal villus length of fish (E) in different dietary chitosan groups at P *<* 0.05 (Statistix 10). Scale bar = 100 μm; image 4x; H & E 200.

Interestingly, the qualitative histological study showed that the three doses of chitosan-treated feed remarkably increased the surface area of the intestine of juvenile *B*. *gonionotus* compared to that of the control fish (Fig 1A–1D). Similarly, the quantitative study revealed that dietary chitosan significantly (P < 0.05) enhanced the length (μm) of the intestinal villi of juvenile *B*. *gonionotus*. Notably, the highest length of intestinal villi of *B*. *gonionotus* was recorded in T1 fish compared to that in the control and other treatments (P < 0.05) (Fig 1E).

### Dietary chitosan enhances lactic acid bacteria in the gut of *B*. *gonionotus*

**Fig 2:**
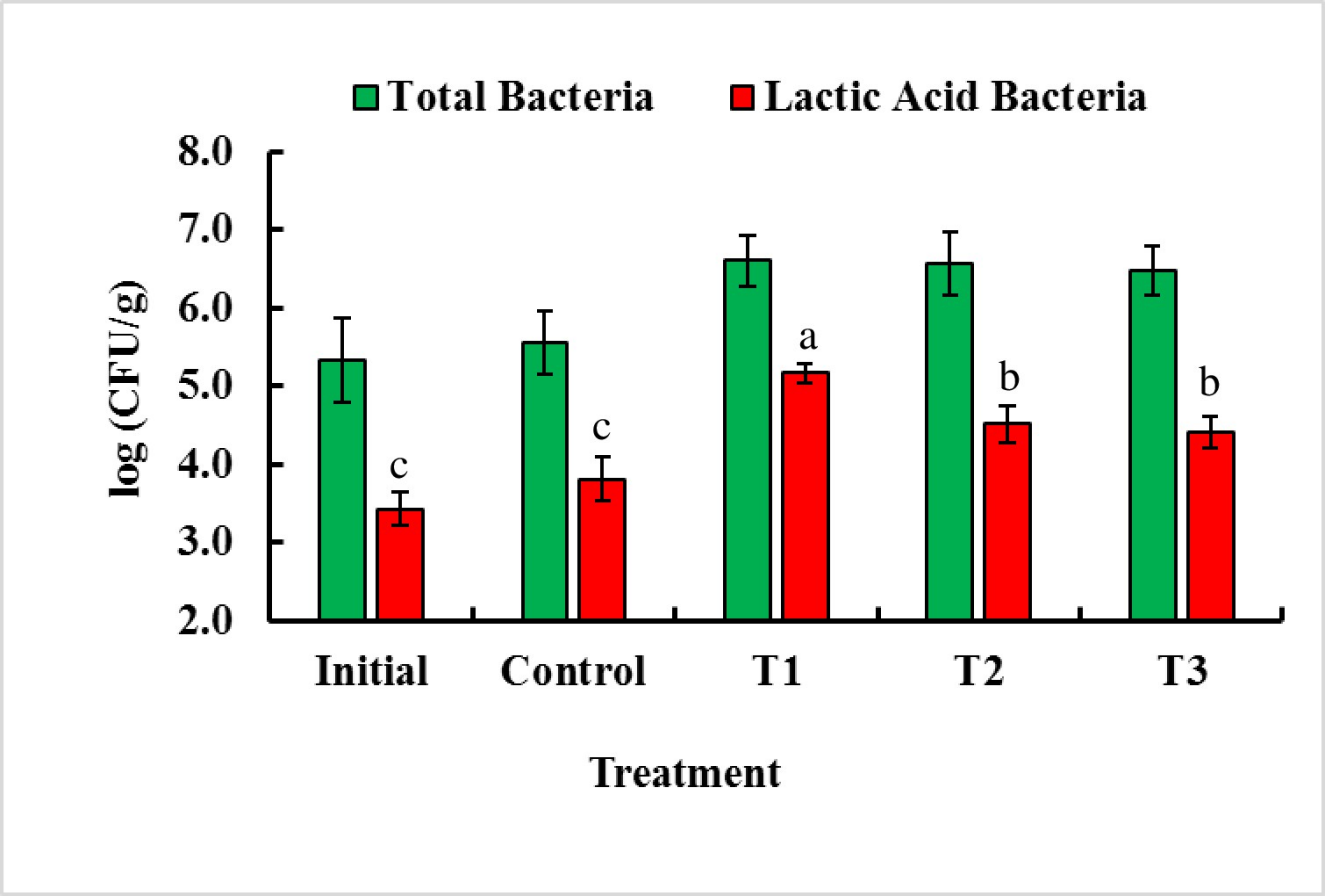
Assessment of the effects of dietary chitosan on the culturable autochthonous bacteria and autochthonous lactic acid bacteria levels (log CFU/g intestine) of *B*. *gonionotus*. One-way ANOVA was performed to analyze the data of three replicate experiments, and the data in the columns varied significantly according to the least significant difference (LSD) at P < 0.05 (Statistix 10). Different letter bars indicate significant variations in the levels of the culturable autochthonous bacteria and autochthonous lactic acid bacteria of *B*. *gonionotus* in different groups at P < 0.05 (Statistix 10). Error bar = ±SD; n = 9.

The highest number of lactic acid bacteria was log (5.63±0.23) CFU g^-1^, log (5.31 ± 0.2) CFU g^-1^, and log (5.02 ± 0.19) CFU g^-1^ in the T1, T2, and T3 groups, respectively, and the lowest number of lactic acid bacteria was log (3.98±0.28) CFU g^-1^ in the control group (Fig 2). Interestingly, dietary chitosan at 1 g kg^-1^ feed (T1) significantly (P < 0.05) enhanced the growth of lactic acid bacteria in the gut of *B*. *gonionotus* compared to that in the T2, T3, or control groups (Fig 2).

### Inhibitory effects of dietary chitosan on the growth of fish pathogenic bacteria *in vitro*

To assess the effects of chitosan on the *in vitro* growth of pathogenic bacteria, a total of four laboratory pathogenic bacterial strains were considered. Interestingly, compared to the control, dietary chitosan at 1 g kg^-1^ feed remarkably inhibited the growth of pathogenic bacteria such as *Aeromonas veronii* B55, *A*. *veronii* Aero1, *Enterococcus faecalis* F1B1, and *E*. *faecalis* PS6 (Table 4).

**Table 4:** Effects of graded levels of dietary chitosan on the growth of fish pathogenic bacterial strains collected from the laboratory. Values are the mean ± SEM (N = 3).

Fish pathogenic bacteria	C1 (CFU ml^-1^)	T4 (CFU ml^-1^)	T5 (CFU ml^-1^)
*Aeromonas veronii* B55^1^	(2.9±0.047)×10^7^	(2.5±0.3)×10^6^	(1.5±0.12)×10^4^
*A*. *veronii* Aero1^1^	(2.9±0.026)×10^7^	(2.76±0.04)×10^6^	(1.78±0.06)×10^3^
*Enterococcus faecalis* F1B1^1^	(3.0±0.08)×10^5^	(2.43±0.34)×10^5^	(1.83±0.09)×10^3^
*E*. *faecalis* PS6^1^	(2.93±0.04)×10^5^	(2.03±0.38)×10^5^	(1.53±0.25)×10^3^

^1^Laboratory strains.

C1 = control (without DMSO).

T4 = DMSO-treated broth culture.

T5 = mixture of DMSO and chitosan-treated broth culture.

### Effects of dietary chitosan on the activities of the intestinal digestive enzymes of juvenile *B*. *gonionotus*

**Fig 3:**
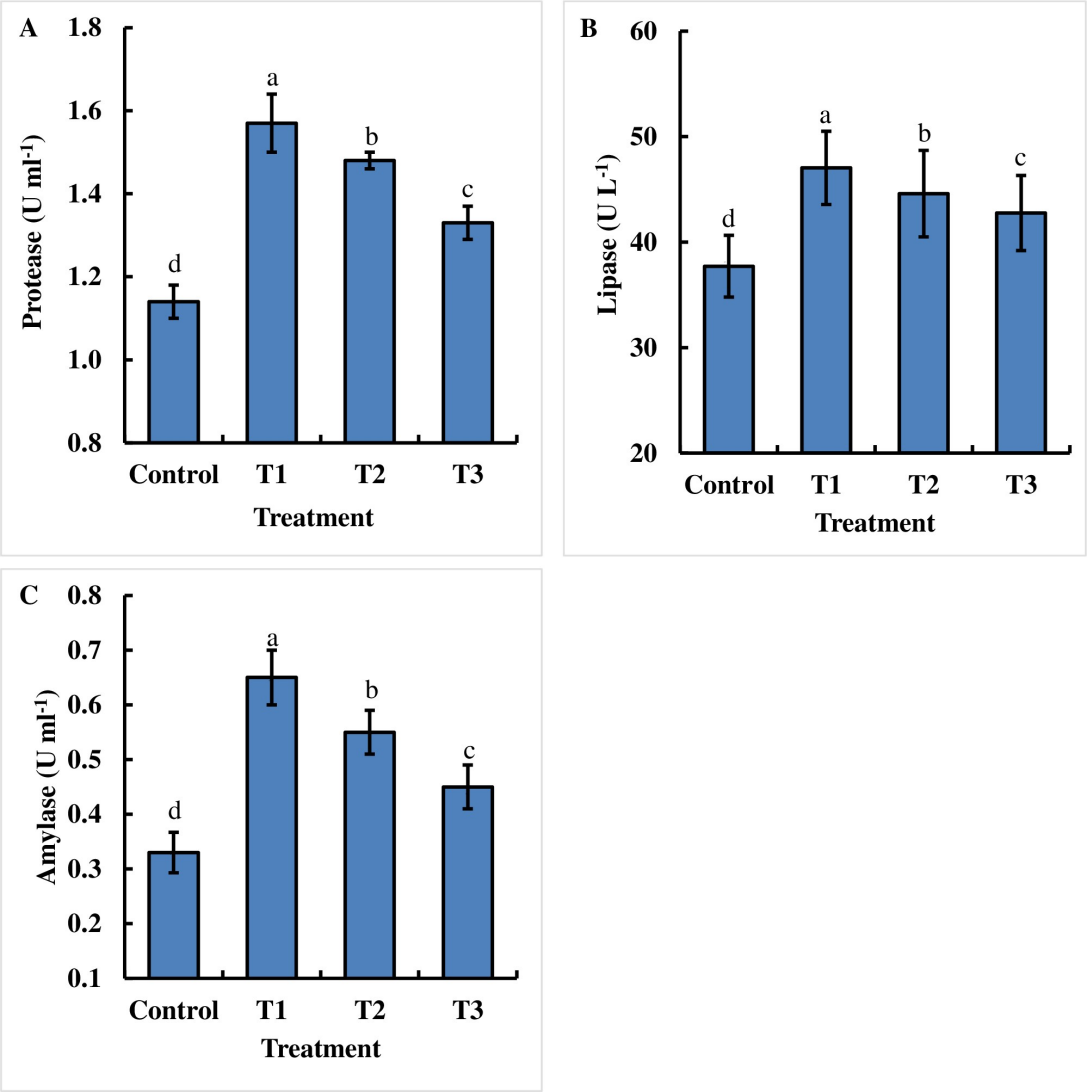
Assessment of the effects of dietary chitosan on the intestinal digestive enzymes (A, B and C) of fish. Fish were reared with three doses of dietary chitosan for 60 days. Here, Control = 0 g kg^-1^ feed; T1 = 1 g kg^-1^ feed; T2 = 2 g kg^-1^ feed and T3 = 3 g kg^-1^ feed. One-way ANOVA was performed to analyze the data of three replicate experiments, and the data in the columns varied significantly according to the least significant difference (LSD) at P < 0.05 (Statistix 10). Different letter bars indicate significant variations in (A) protease, (B) lipase, and (C) amylase levels of the fish in different dietary chitosan groups at P < 0.05 (Statistix 10). Error bar = ±SD; n = 9.

The dietary chitosan-treated formulated feed significantly (P < 0.05) increased the activities of intestinal digestive enzymes such as protease, lipase, and amylase in the gut of *B*. *gonionotus* (Fig 3). Interestingly, the dietary chitosan at a lower dose at 1 g kg^-1^ feed exhibited the significantly highest intestinal digestive enzyme activities in the gut of *B*. *gonionotus* compared to those with the control and other treatments (Fig 3).

### Effects of dietary chitosan on the proximate body composition of juvenile *B*. *gonionotus*

**Fig 4:**
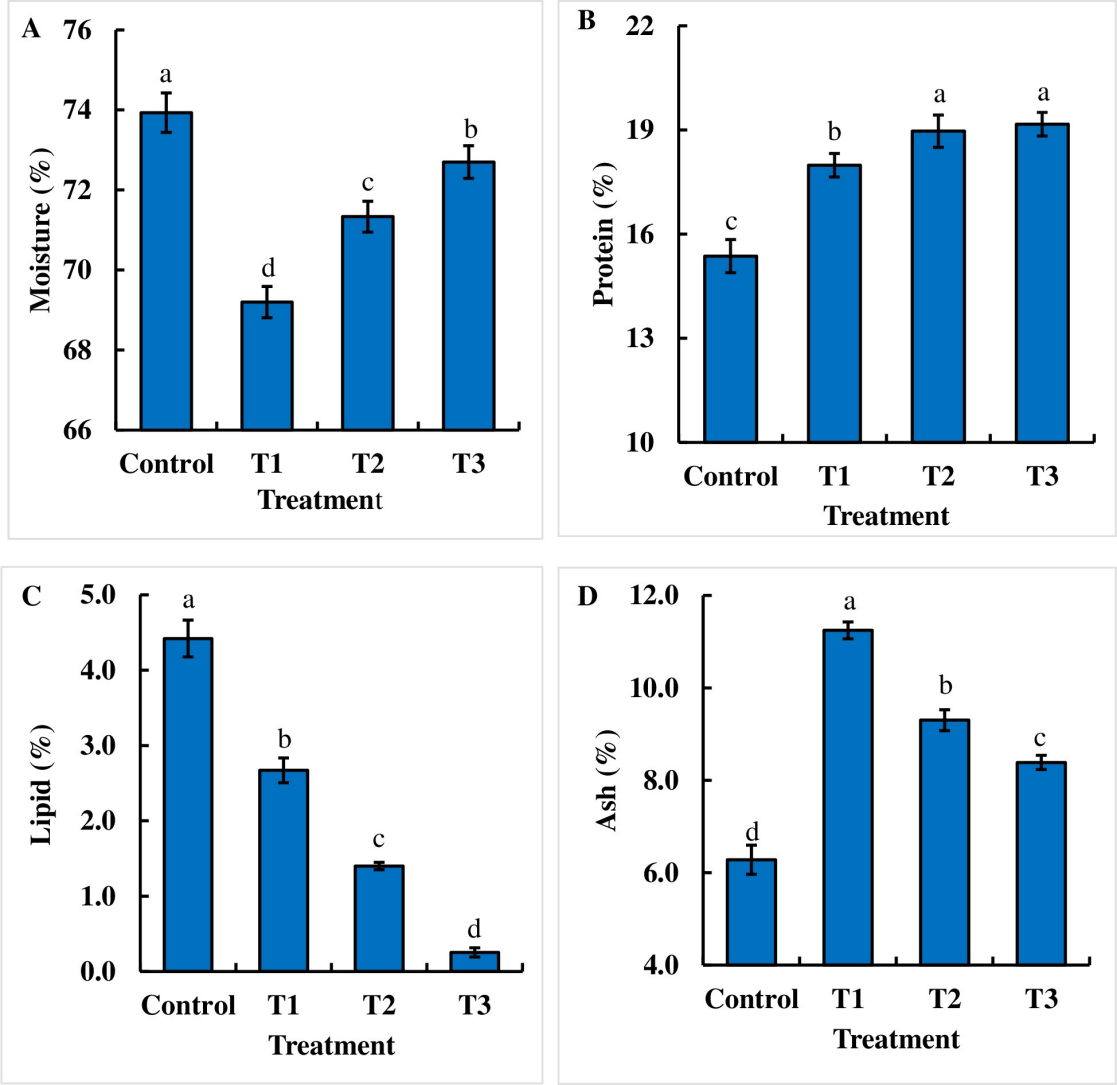
Assessment of the effects of dietary chitosan on the proximate body composition (A, B C and D) of fish. Fish were reared with three doses of dietary chitosan for 60 days. Here, Control = 0 g kg^-1^ feed; T1 = 1 g kg^-1^ feed; T2 = 2 g kg^-1^ feed and T3 = 3 g kg^-1^ feed. One-way ANOVA was performed to analyze the data of three replicate experiments, and the data in the columns varied significantly according to the least significant difference (LSD) at P < 0.05 (Statistix 10). Different letter bars indicate significant variations in (A) moisture (%), (B) protein (%), (C) lipid (%), and (D) ash (%) contents of the fish in different dietary chitosan groups at P < 0.05 (Statistix 10). Error bar = ±SD; n = 9.

Dietary chitosan-treated feed significantly decreased the moisture content (%) compared to that in the control group (Fig 4A). Interestingly, the significantly lowest moisture content (%) was recorded in the T1 group compared to that in the control and other treatment groups (Fig 4A). Interestingly, the protein content (%) was significantly (P < 0.05) enhanced in the chitosan-treated feed groups compared to that in the control group, and the increase in the doses of dietary chitosan increased the protein content (%) in fish (Fig 4B). Dietary chitosan-treated feed significantly decreased the lipid content (%) compared to that with the control (Fig 4C). The significantly lowest lipid content (%) was recorded in the T3 group compared to that in the control and other treatment groups (Fig 4C). Conversely, the lipid content (%) was significantly (P < 0.05) lower with the diets with increased dietary chitosan levels than with the control diet (Fig 4C). Moreover, the dietary chitosan-treated formulated feeds significantly (P < 0.05) increased the ash content (%) in the carcass compared to that with the control (Fig 4D). Interestingly, the significantly highest ash content (%) was recorded in the T1 group compared to that in the control and other treatment groups (Fig 4D).

### Dietary chitosan-treated feeds modulate the mineral contents in the muscles of *B*. *gonionotus*

There were linear and quadratic expanding patterns in mineral substance, like those of Na (%), K (%), Fe (%), Zn (%), and Mn (%) in the muscles of *B*. *gonionotus* fed chitosan-supplemented diets, although the differences were not significant (Table 5). Similarly, according to ANOVA, dietary chitosan produced no significant change in mineral contents, such as those of Na (%), K (%), Fe (%), Zn (%), and Mn (%), in the muscles of *B*. *gonionotus* compared to those with the control (Table 5). However, the muscle composition of *B*. *gonionotus* showed significant (P < 0.05) linear trends for Ca (%) and Mg (%) contents in the muscles. The muscle composition of *B*. *gonionotus* showed a significant (P < 0.05) quadratic trend for Ca (%) but not Mg (%) contents in response to dietary chitosan supplementation (Table 5). Similarly, dietary chitosan significantly (P < 0.05) reduced Ca (%) content and significantly (P < 0.05) increased Mg (%) content in fish muscles compared to those with the control (Table 5).

**Table 5:** Mineral composition of the muscle of *B*. *gonionotus* fed diets with graded levels of dietary chitosan after a 2-month feeding trial. Values are the mean ± SEM (N = 3).

Parameter	Diet designation (g kg^-1^ feed)				Pr > F^1^		
	Control (0)	T1 (1.0)	T2 (2.0)	T3 (3.0)	ANOVA	Linear trend	Quadratic trend
Na (%)	0.712±0.003	0.713±0.004	0.716±0.002	0.719±0.004	0.148	0.331	0.595
K (%)	1.46±0.027	1.47±0.016	1.44±0.021	1.42±0.029	0.156	0.064	0.231
Ca (%)	1.47±0.025	1.44±0.031	1.26±0.029	1.21±0.029	0.000	0.04	0.02
Mg (%)	0.37±0.014	0.38±0.005	0.49±0.013	0.50±0.011	0.000	0.000	0.775
Fe (%)	0.027±0.0009	0.027±0.007	0.027±0.0018	0.027±0.0008	0.715	0.982	0.276
Zn (%)	0.094±0.003	0.094±0.003	0.093±0.002	0.091±0.003	0.409	0.141	0.467
Mn (%)	0.034±0.003	0.037±0.003	0.038±0.002	0.037±0.003	0.660	0.303	0.521

^1^ Significance probability associated with the F-statistic.

### Assessment of hematological parameters in dietary chitosan-treated *B*. *gonionotus*

**Fig 5:**
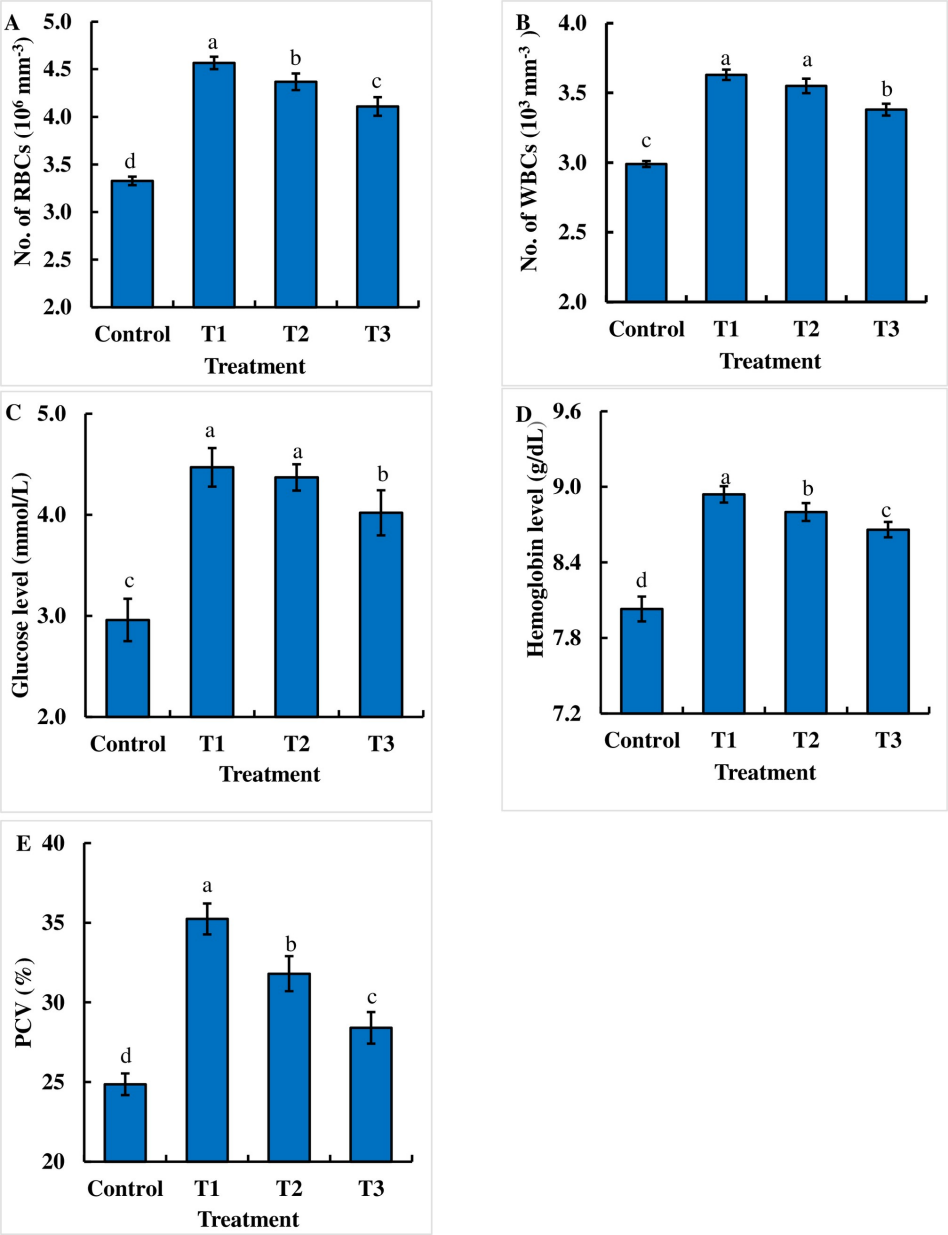
Assessment of the effects of dietary chitosan on the hematological parameters (A, B, C, D and E) of fish. Blood was collected from thirty fish of each replication and 90 fish of each treatment for estimating the (A) red blood cell (RBC) count, (B) white blood cell (WBC) count, (C) glucose level, (D) hemoglobin level and (E) packed cell volume (PCV). One-way ANOVA was performed to analyze the data of three replicate experiments, and the data in the columns varied significantly according to the least significant difference (LSD) at P < 0.05 (Statistix 10). Different letter bars indicate significant variations in the RBC count, WBC count, glucose level, hemoglobin level and PCV (%) of the fish in different dietary chitosan groups at P < 0.05 (Statistix 10). Error bar = ±SD; n = 90.

The dietary chitosan-treated formulated feed significantly (P < 0.05) increased the hematological parameters in terms of RBCs, WBCs, glucose level, hemoglobin level, and PCV (%) compared to those with the control (Fig 5). Interestingly, RBCs, WBCs, the glucose level, the hemoglobin level, and PCV (%) were significantly (P < 0.05) higher in fish fed feed supplemented with dietary chitosan at a dose of 1 g kg^-1^ feed than in fish fed feed without dietary chitosan (Fig 5).

### Effects of dietary chitosan-treated feed on the morphology of the kidney and liver of *B*. *gonionotus*

**Fig 6:**
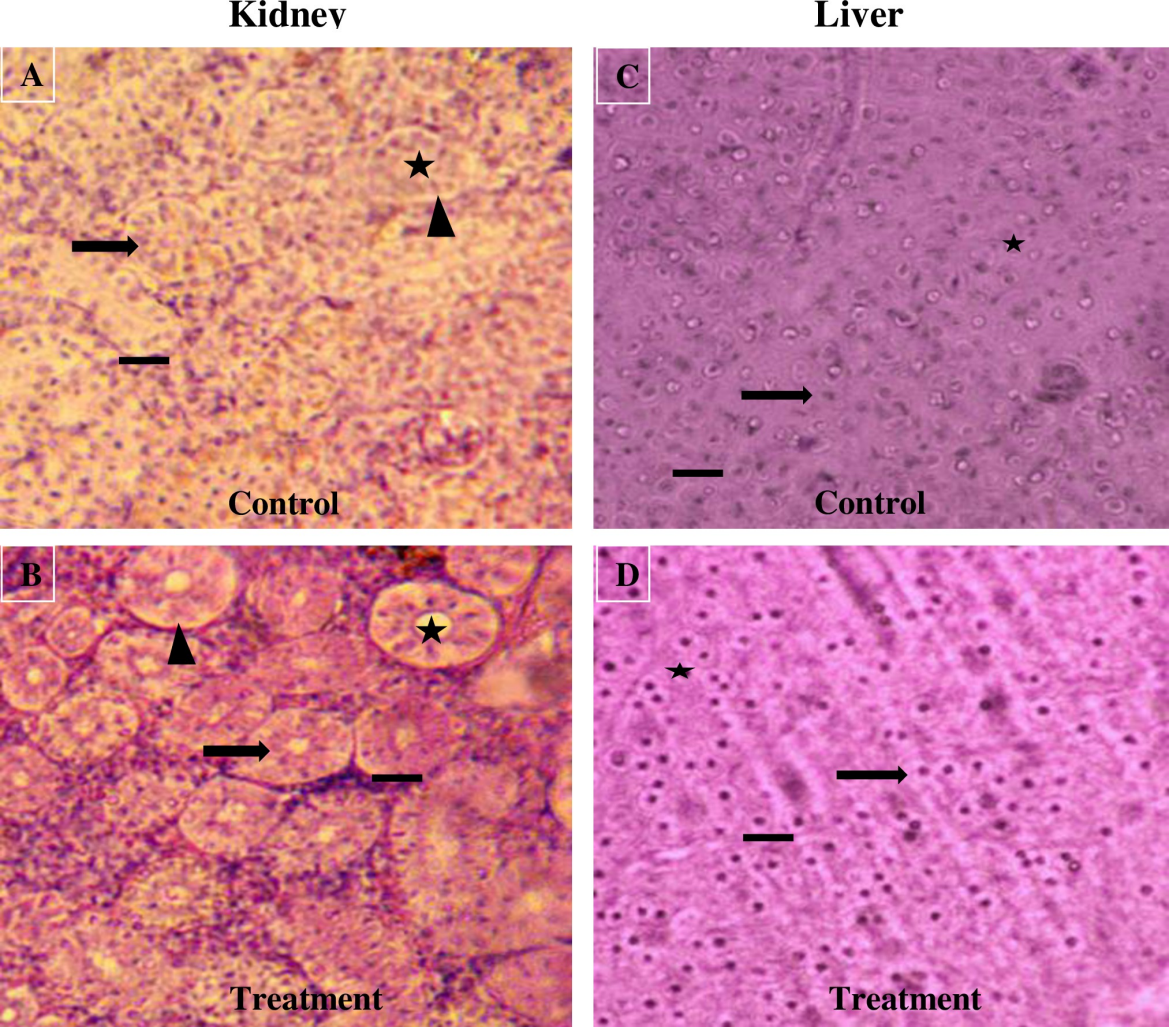
Histological analysis of the effects of dietary chitosan on the kidneys (A and B) and liver (C and D) of *B*. *gonionotus*. Fish were fed a (A and C) control diet (chitosan at 0 kg^-1^ feed) or dietary chitosan at 1 g kg^-1^ (B and D). (A) shows an unclear renal corpuscle (arrow), glomerulus (asterisk), Bowman’s space (arrowhead) and proximal tubules. (B) represents a more well-defined renal corpuscle, glomerulus, Bowman’s space and proximal tubules than A. (C) shows unclear hepatocytes (arrow) with irregularly shaped nuclei (asterisk). (D) shows hepatocytes with regularly shaped nuclei. Six fish were considered for each treatment. Image 40×; scale bar = 50 μm; H & E 200; n = 9.

After histological processing, the liver and kidney slides were imaged under a light microscope to observe the effects of the dietary chitosan treatments (chitosan at 1 g kg^-1^) on the morphology of the liver and kidney of the fish (Fig 6). Qualitative histological study revealed that the dietary chitosan-treated diet remarkably improved the morphology of kidneys and significantly reduced the amount of fat bodies in the kidney. Similarly, dietary chitosan also improved the morphology of the liver of *B*. *gonionotus* compared to that with the control (Fig 6).

## Discussion

Chitosan is a biodegradable, biocompatible and safe biopolymer. In the present study, the application of dietary chitosan significantly improved the growth, biochemical composition of the whole body, gut microbiota, activities of intestinal digestive enzymes, hematological parameters and histology of the intestine, liver, and kidney of juvenile *B*. *gonionotus*. Although the enhancement of growth, innate immunity, hematological parameters, nutrient and mineral contents, and lactic acid bacteria of fish treated with dietary chitosan have been reported by several researchers [11–14,26], this study demonstrated for the first time that the optimum level of dietary chitosan significantly improved the growth, biochemical composition, gut microbiota, digestive enzyme activities, histology, and hematological parameters of *B*. *gonionotus*, likely suppressing the growth of gut pathogenic bacteria.

Oral administration of dietary chitosan has been shown to increase the growth performance of weaned pigs [47], tiger puffers [48], sea cucumbers [49], Japanese quail [50], turbot [51], Japanese koi [52], and loaches [26]. In the current work, the dietary chitosan-treated formulated feed positively improved body weight gain, %BWG, and SGR compared with those of the control group, while FCR exhibited the opposite trend. These results might imply that the oral administration of dietary chitosan increased the activities of intestinal digestive enzymes [26]. In the current study, dietary chitosan tremendously enhanced the protease, lipase, and amylase activities in the gut of *B*. *gonionotus*. These digestive enzymes might be answerable for the hydrolysis of the significant supplements to hydrolysates that might be absorbed in the digestive system. However, the administration of a high level of dietary chitosan decreased growth parameters such as body weight gain, %BWG and SGR values, and increased the FCR compared with those with a moderate dose of chitosan, which is supported by many researchers [15,17,26]. The high concentration of dietary chitosan might mainly induced hypolipidemic activity [53].

Although the optimal level of chitosan (1 g kg^-1^ feed) significantly enhanced growth compared to that with the high doses of dietary chitosan in the current study, the high level of dietary chitosan (3 g kg^-1^ feed) tremendously increased the essential nutrient protein (%) and ash contents (%) compared to those with the lower doses and the control. We also found that there was an inverse relationship between protein content (%) and lipid content (%) in the body proximate composition of *B*. *gonionotus*. Consequently, the high-level chitosan drastically decreased the lipid content (%), which is not enough for human health, although the fish exhibited a high level of protein content (%). Similar results have been found in mammals such as pigs [54] and mice [55]. Zaki et al. [15] reported that a lower level of chitosan (1 g kg^-1^) significantly increased the protein content (%) and decreased the lipid content (%) compared with those of the control, which is consistent with the results of the present study. Although the mechanisms of the effects of chitosan on fish are not clear from our data, the findings of the present study imply that dietary chitosan may bind with monounsaturated fatty acids and polyunsaturated fatty acids (18: 2n-6) of freshwater fishes [21]. On the other hand, the high level of dietary chitosan produced no change in lipid content (%) in marine and saltwater fishes that contain highly polyunsaturated fatty acids (20:2n-3) [20,23]. In our study, the lower level of dietary chitosan significantly increased the ash content (%) compared to that with the moderate and higher doses as well as the control, which is contradictory to the ash content reported by Zaki et al. [15]. Dietary chitosan-incorporated diets did not significantly affect essential mineral contents, such as those of Na (%), K (%), Fe (%), Zn (%), and Mn (%), in the muscle of *B*. *gonionotus*. Similarly, Tosun et al. [22] reported that dietary chitosan produced no change in mineral content and dissolution in the inorganic content in a tooth of humans, which is in agreement with the present study. In contrast, there was an inverse relationship between Ca and Mg contents (%) in the muscles when fish were fed chitosan-treated feed. We found that moderate and high levels of dietary chitosan decreased the Ca content (%) and increased the Mg content (%) in the muscles, respectively. Based on these results, it is best to apply dietary chitosan at a dose of 1 g kg^-1^ feed to maintain the mineral and nutrient balance in the body. Likewise, Deuchi et al. [56] showed that continuous and massive intake of dietary chitosan affected the mineral and fat-soluble vitamin status in rats.

Intestinal morphology plays a role in the physiology of nutrient absorption and metabolism [57]. In the current study, the oral administration of dietary chitosan significantly enhanced the length of intestinal villi, and the dietary chitosan-treated diet at 1 g kg^-1^ feed produced an outstanding increase in the length of villi of *B*. *gonionotus*. Najafabad et al. [23] reported that the chitosan level of a 1 g kg^-1^ diet significantly enhanced the length of villi of *Caspian kutum*, which is consistent with the present study. These results suggest that the increased length of villi might be able to increase the intestinal nutrient absorption area, which might contribute to an improvement in feed utilization and growth. However, the mechanism by which dietary chitosan improves the intestinal absorption of fishes is not clear. By and large, the cells on the tip of these villi are consistently sloughed off, and the reestablishment of the intestinal epithelium is accounted for to be exceptionally high to replace these cells [58]. In the current study, dietary chitosan likely modulated the intestinal microbiota by reducing the number of pathogenic bacteria, which ultimately promoted healthy intestinal villi [58]. This mechanism may be because dietary chitosan can inhibit the growth of pathogenic microbes in the gut of fish, and gut probiotic bacteria can then obtain the opportunity to secrete byproducts that can activate digestive enzymes for digestion in the gut of fish. Consequently, dietary chitosan might increase the length of the intestinal villi in addition to the renewal of intestinal epithelial cells. A previous study applying dietary prebiotics, including inulin and Jerusalem artichoke, also reported changes in the intestinal microbiota that were related to the length of intestinal villi in Nile tilapia [59]. Similarly, we found that dietary chitosan significantly enhanced the abundance of lactic acid bacteria (LABs) in the intestine compared to that with the control in the present study. Clearly, dietary chitosan showed a positive relationship with other lactic acid bacteria in the host gut. These LABs have beneficial effects on the gut microbiome and inhibit the growth of various fish pathogens [26,41,60,61]. This phenomenon is presumably due to the antibacterial activity of dietary chitosan [26], which inhibited the growth of pathogenic bacteria.

Assessment of hematological parameters helps to interpret the health status of fishes. In the present study, significant positive changes in response to dietary chitosan were observed among the hematological parameters tested during the experimental period. We investigated the effects of dietary chitosan as a natural substance on the hematological parameters of juvenile *B*. *gonionotus*. Coincidentally, supplementation with dietary chitosan led to increased hematological parameters in *Carassius auratus gibelio* [16]. Dietary chitosan might enhance the RBC, sugar, and hemoglobin levels in the blood; consequently, dietary chitosan-treated fish might receive a sufficient amount of oxygen for respiration and high metabolic activity [62,63]. Meshkini et al. [24] reported that dietary chitosan at 0.25% showed positive effects on hematological indices, which is consistent with the findings of the present study. However, in some cases, dietary chitosan did not show any effect on RBCs, hemoglobin, PCV (%), or sugar levels of fishes [23]. These results suggest that dietary chitosan may improve the cardiovascular health of fishes. Moreover, the dietary chitosan used in the current study increased the amount of WBCs, which might enhance the immunity of fish, resulting in the prevention of fish diseases [64]. Similarly, supplementation with dietary chitosan increased the number of WBCs in *C*. *auratus gibelio* and *Rutilus frisii kutum* [16,23]. Feeding with dietary chitosan led to a significant increase in the number of erythrocytes and WBCs, which might help in nonspecific immunity via neutrophils and macrophages [65]. Taken together, these results suggest that the effects of dietary chitosan on hematological parameters may vary among fish species and with the concentration of dietary chitosan.

Dietary chitosan may reduce fat accumulation in the liver, which accumulates from formulated feed ingredients, including soybean meal and mustard oil cake. These results indicate that the dietary chitosan-treated formulated feeds would be safe for fish and beneficial for human health. Microscopic observations revealed that there were normal structures in the liver and kidney after dietary chitosan supplementation. Strangely, the dietary chitosan-treated defined eating regimens in the current examination kept up with ordinary capacity of all parts of the kidney of *B*. *gonionotus*. Thilagar and Samuthirapandian [19] reported that dietary chitosan-treated feed improved the morphology of the intestine, liver, and kidney of *Oreochromis mossambicus*. Moreover, there were no significant changes in morphology in fish fed chitosan alone, which indicated the nontoxic nature of dietary chitosan for fish [66]. These results suggest that dietary chitosan supplemented in formulated diets is safe for sustainable aquaculture.

## Conclusion

In conclusion, the dietary chitosan-treated formulated feeds (1 g kg^-1^) promoted growth and nutrient composition in the muscles, the gut microbiota, digestive enzyme activities, hematological parameters, and intestinal, liver, and kidney morphology of juvenile *B*. *gonionotus* fishes. However, dietary chitosan had no detectable effects on mineral contents in the muscles of *B*. *gonionotus*. Our findings suggest that dietary chitosan could be supplemented at a dose of 1 g kg^-1^ feed to a practical diet to enhance the growth and development of cultured *B*. *gonionotus*. Further study is warranted to elucidate the precise mechanisms of the positive effects of dietary chitosan on juvenile *B*. *gonionotus*.

## Supporting information

S1 File ARRIVE Essential 10. (DOCX)

S1 Table Exact feed consumed by fish and nutrient composition of that feed. (DOCX)
