# Supplementary material for: Dietary chitosan promotes the growth, biochemical composition, gut microbiota, hematological parameters and internal organ morphology of juvenile Barbonymus gonionotus
Source: PLoS One. 2021 Nov 18;16(11):e0260192. doi: 10.1371/journal.pone.0260192 (PMC8601453; doi:10.1371/journal.pone.0260192)
Supplement: S1 File — (DOCX) [file pone.0260192.s001.docx]

**Supplementary materials for**

**Dietary chitosan promotes the growth, biochemical composition, gut microbiota, hematological parameters and internal organ morphology of juvenile *Barbonymus gonionotus***

**Mohammad Abdus Salam^1^*, Md. Ashikur Rahman^1^, Sulav Indra Paul^2^, Fatama Islam^1^, Avishek Kanti Barman^1^, Zinia Rahman^1^, Dinesh Chandra Shaha^3^, Md. Mahbubur Rahman^2^, Tofazzal Islam^2^**

^1^ Department of Genetics & Fish Breeding, Faculty of Fisheries, Bangabandhu Sheikh Mujibur Rahman Agricultural University, Gazipur-1706, Bangladesh.

^2^ Institute of Biotechnology and Genetic Engineering, Bangabandhu Sheikh Mujibur Rahman Agricultural University, Gazipur-1706, Bangladesh.

^3^ Department of Fisheries Management, Bangabandhu Sheikh Mujibur Rahman Agricultural University, Gazipur-1706, Bangladesh.

*Corresponding author

Email: [salamfish@bsmrau.edu.bd](mailto:salamfish@bsmrau.edu.bd)

**ARRIVE Essential 10**

**Item 1. Study design**

**For each experiment, provide brief details of study design including:**

**1a. The groups being compared, including control groups. If no control group has been used, the rationale should be stated.**

**Statement:** To assess the effect of dietary chitosan in formulated feed, twelve plastic tanks (500L) were divided into four treatments *viz*., control, T1, T2 and T3. Each treatment has three replicates. The control treatment was without supplementation of chitosan (0 g kg^-1^ feed) in formulated feed. Whereas T1, T2 and T3 treatments were supplemented with addition of three concentrations of chitosan at the doses of 1g kg^-1^, 2g kg^-1^ and 3g kg^-1^, respectively. A total of 360 uniformly sized juvenile fish were randomly distributed in four treatments and the stocking density was maintained at 30 fish/tank following a completely randomized design. The fishes were acclimatized with commercial feeds for 15 days. After the acclimatization period, control treatment was fed without the dietary chitosan, and treatments T1, T2, and T3 were fed with addition of three concentrations of chitosan at the doses of 1g kg^-1^, 2g kg^-1^ and 3g kg^-1^, respectively for an experimental period of 60 days.

Fish hand-fed daily at 6% of the total biomass, twice daily at 0900 h and 1900 h for a period of 60 days. Feed adjustments were done for each tank every 15 days after sampling. The dough was prepared every 7 days interval. The uneaten feeds were collected during water exchange. During the experiment, the uneaten feed were collected regularly to calculate the exact feed intake by experimental fish. Treatment wise feed consumption percentages were control (80%), T1 (87%), T2 (85%) and T3 (83%).

**1b. The experimental unit (e.g., a single animal, litter, or cage of animals).**

**Statement:** A total of 360 uniformly sized juvenile fish were randomly distributed in four treatments and the stocking density was maintained at 30 fish/tank following a completely randomized design.

**Item 2. Sample size**

**2a. Specify the exact number of experimental units allocated to each group, and the total number in each experiment. Also indicate the total number of animals used.**

**Statement:**

- A total of 360 uniformly sized fish were randomly distributed in four treatments and the stocking density was maintained at 30 fish/tank following a completely randomized design.
- For the measurement of growth parameter, a total of 90 fish (each replication contain 30 fish) were taken for each treatment.
- Nine fishes from each treatment were used for chemical analysis of carcass composition of *B. gonionotus*.
- For the analysis of mineral contents in muscles of *B. gonionotus* nine fishes from each treatment were used.
- At the end of the feeding trial of growth performances, 18 fish from each treatment (6 fish from each replication) by random sampling were examined to assess the effect of the dietary chitosan on gut microbiota.
- Nine fish from each treatment were randomly sampled to assay the intestinal digestive enzyme activity of *B. gonionotus*.
- For histological study, nine fish from each treatment were used.
- For hematological analysis, 90 fish from each treatment were used.

**2b. Explain how the sample size was decided. Provide details of any a priori sample size calculation, if done.**

**Statement:** Power analysis was performed to check the statistical validity of sample size. The typical power analysis for an ANOVA was performed using G*Power version 3.0.10.

**Item 3. Inclusion and exclusion criteria**

**3a. Describe any criteria used for including or excluding animals (or experimental units) during the experiment, and data points during the analysis. Specify if these criteria were established a priori. If no criteria were set, state this explicitly.**

**Statement:** During the experiment, no experimental animals were included or excluded.

**3b. For each experimental group, report any animals, experimental units, or data points not included in the analysis and explain why. If there were no exclusions, state so.**

**Statement:** For each experimental group, all the data were included in the statistical analysis.

**3c. For each analysis, report the exact value of *n* in each experimental group.**

**Statement:** Value of *n* in each experimental group are given below:

1. For the measurement of growth parameter: *n = 90*.
2. For the analysis of carcass composition: *n = 9*.
3. For the analysis of mineral contents in muscles: *n = 9*.
4. To assess the effect of the dietary chitosan on gut microbiota: *n = 18*.
5. To assay the intestinal digestive enzyme activity: *n = 9*.
6. For histological study: *n = 9*.
7. For hematological analysis: *n = 90*.

**Item 4. Randomisation**

**4a. State whether randomisation was used to allocate experimental units to control and treatment groups. If done, provide the method used to generate the randomisation sequence.**

**Statement:** All the experimental animals were randomly distributed in treatment groups by completely randomized design using a computer based random order generator

**4b. Describe the strategy used to minimise potential confounders such as the order of treatments and measurements, or animal/cage location. If confounders were not controlled, state this explicitly.**

**Statement:** Test time was between twice daily at 0900 h and 1900 h for a period of 60 days, and testing order was randomized daily, with each animal tested at a different time each test day.

**Item 5. Blinding**

**Describe who was aware of the group allocation at the different stages of the experiment (during the allocation, the conduct of the experiment, the outcome assessment, and the data analysis).**

**Statement:** Personnel who analyze the data collected from the study are not aware of the treatment applied to any given group. Investigation: Md. Ashiqur Rahman, Fatama Islam, Sulav Indra Paul, Avishek Kanti Barman, Mohammad Abdus Salam. Formal analysis: Mohammad Abdus Salam, Fatama Islam.

**Item 6. Outcome measures**

**6a. Clearly define all outcome measures assessed (e.g., cell death, molecular markers, or behavioural changes).**

**Statement:** The following parameters were assessed: growth performances, analysis of carcass composition and mineral composition, histological and hematological parameters, the effect of the dietary chitosan on gut microbiota and intestinal digestive enzyme activity of *B. gonionotus*

**6b. For hypothesis-testing studies, specify the primary outcome measure, i.e., the outcome measure that was used to determine the sample size.**

**Statement:** The primary outcome of this study is to assess the effects of dietary chitosan on growth and development of fish.

**Item 7. Statistical methods**

**7a. Provide details of the statistical methods used for each analysis, including software used.**

**Statement:** All data (body weight gain, weight gain, SGR, %BWG, FCR, moisture, protein, lipid and ash and minerals of body carcass, blood parameter and gut microbiota) were collected during the study period and statistically analysed using one-way analysis of variance (ANOVA) to test the significant results (P < 0.05) between means and the mean values were separated by LSD (least significance difference) posthoc statistic. Standard deviation (±SD) was calculated to identify the range of means. Minerals of body carcass of fish statistically analyzed using one-way analysis of variance (ANOVA), and linear and quadric trends to test the significance of the experimental results (P < 0.05) between means. All statistical analyses were performed with the aid of the computer software Statistix 10.0 version. Power analysis was performed to check the statistical validity of sample size. The typical power analysis for an ANOVA was performed using G*Power version 3.0.10.

**7b. Describe any methods used to assess whether the data met the assumptions of the statistical approach, and what was done if the assumptions were not met.**

**Statement:** The typical power analysis for an ANOVA was performed using G*Power version 3.0.10 to check the statistical validity of data and found all the data statistically valid.

**Item 8. Experimental animals**

**8a. Provide species-appropriate details of the animals used, including species, strain and substrain, sex, age or developmental stage, and, if relevant, weight.**

**Statement:** A total of 360 (average 20.9 ± 0.56 g) juvenile experimental fish (*B. gonionotus*) were used in this experiment.

**8b. Provide further relevant information on the provenance of animals, health/immune status, genetic modification status, genotype, and any previous procedures.**

**Statement:** Healthy *B. gonionotus* fish were collected from Bismillah fish hatchery from Trishal, Mymensingh, Bangladesh.

**Item 9. Experimental procedures**

**For each experimental group, including controls, describe the procedures in enough detail to allow others to replicate them, including:**

**9a. What was done, how it was done, and what was used.**

**Statement:** Detail protocols are available at protocols.io website ([dx.doi.org/10.17504/protocols.io.bt2knqcw](https://dx.doi.org/10.17504/protocols.io.bt2knqcw) )

**9b. When and how often.**

**Statement:** Fish hand-fed daily at 6% of the total biomass, twice daily at 0900 h and 1900 h for a period of 60 days. Feed adjustments were done for each tank every 15 days after sampling. The dough was prepared every 7 days interval. The uneaten feeds were collected during water exchange. Water quality parameters such as, pH, dissolve oxygen, and temperature were routinely measured to maintain the health of fish.

**9c. Where (including detail of any acclimatisation periods).**

**Statement:** The fish were stocked in the circular plastic tanks (500L) with aerators and acclimatized for 15 days in the wet laboratory. Water change was done every 3 days interval and uneaten feeds were collected.

**9d. Why (provide rationale for procedures).**

**Statement:** Not applicable in this research.

**Item 10. Results**

**For each experiment conducted, including independent replications, report:**

**10a. Summary/descriptive statistics for each experimental group, with a measure of variability where applicable (e.g., mean and SD, or median and range).**

**Statement:** Mean ± SD values are presented below:

1. In the case of a chitosan application, the weight gains of fishes were 26.11 ± 1.40, 39.40 ± 1.47, 34.37± 1.24 and 30.17 ± 0.60 g in control, T1, T2, and T3, respectively after the end of 60 days (Fig. 1A).
2. The food conversion ratio (FCR) in control, T1, T2, and T3 were 1.48 ± 0.022, 1.2 ± 0.029, 1.3 ± 0.020, and 1.38 ± 0.008, respectively after 60 days (Fig. 2E).
3. The highest length of intestinal villi of *B. gonionotus* were recorded in T1 compared to control and other treatments (Fig. 2E).
4. The highest number of lactic acid bacteria were log (5.63±0.23) CFUg^-1^, log (5.31 ± 0.2) CFUg^-1^ and log (5.02 ± 0.19) CFUg^-1^ in T1, T2 and T3, respectively and the lowest number of lactic acid bacteria was log (3.98±0.28) CFUg^-1^ in control (Fig. 3).

**10b. If applicable, the effect size with a confidence interval.**

**Statement:** Power analysis was performed at 95% confidence interval.

**Item 11. Abstract**

**Provide an accurate summary of the research objectives, animal species, strain and sex, key methods, principal findings, and study conclusions.**

**Statement:** This study aimed to determine the effects of dietary chitosan on growth, biochemical composition, hematological and histological parameter by improving the gut microbiota status of juvenile *Barbonymus gonionotus*. Three test diets containing three different levels (1, 2 and 3g kg^-1^) of dietary chitosan were formulated. A basal diet without dietary chitosan considered as control and fish were reared for 60 days. Fish treated with dietary chitosan significantly improved growth, nutrients and mineral contents of the muscles, hematological parameter, lactic acid bacteria and activities of intestine digestive enzymes of *B. gonionotus* compared to control. Interestingly, the increase of chitosan inclusion level in diets significantly enhanced the protein contents and inversely significantly decreased the lipid contents compared to control. The dietary chitosan significantly inhibited the growth of pathogenic bacteria of fish. The quantitative study revealed that chitosan significantly enhanced length of intestinal villi and the qualitative study displayed that chitosan considerably reduced the fat content in liver and improved the morphology of the kidney compared to control. Finally, application of chitosan at 1g kg^-1^ feed showed the highest beneficial effects to the treated *B. gonionotus* indicating their potentials for practical application in safe aquaculture.

**Item 12. Background**

**12a. Include sufficient scientific background to understand the rationale and context for the study, and explain the experimental approach.**

**Statement:** The prebiotic dietary chitosan also exert beneficial effects to the treated fish in aquaculture. Many scientists demonstrated that application of probiotics promoted growth, immune system and hematological parameter and suppressed fish diseases. However, little information is available on the effect of the dietary chitosan from scales of finfish, shells of crustaceans and the cell wall of fungi on growth promotion and health improvement of fishes. A new challenge concerning fish nutrition and disease control look for a new economically viable option that are environment-friendly. In this respect, chitosan may make a significant contribution in safe aquaculture. In this aspect, the present study was conducted i) to elucidate the effect of dietary chitosan on growth, biochemical composition and hematological parameter ii) to assess the effect of dietary chitosan on gut microbiota status and digestive enzymes of juvenile *B. gonionotus* and on pathogenic bacteria of fishes and iii) to analyze the effect of dietary chitosan on the histology of intestine, liver and kidney of juvenile *B. gonionotus*.

**12b. Explain how the animal species and model used address the scientific objectives and, where appropriate, the relevance to human biology.**

**Statement:** World fish production is highly increased to about 171 million tonnes in 2016, where aquaculture represented 47% of the total. In Bangladesh, carp production is about 1.19 million metric tonnes, which is about 32.6% of the total fish production. However, average fish production in aquaculture of Bangladesh is still much lower than many other carp producing countries like China. In this respect, a minor carp, *Barbonymus gonionotus* (Bleeker, 1850), commonly known as silver barb in aquaculture, widely distributed in almost all the countries in the world especially in South East Asian countries for aquaculture. Because of its high popularity, its distribution has been widely extended by human introduction. This species is widely used in polyculture as well as weed control in South East Asia and also important fish species for integrated rice-fish farming. The interest in *B. gonionotus* as a means of biological control of weeds instead of grass carp that destroy the plants.

**Item 13. Objectives**

**Clearly describe the research question, research objectives and, where appropriate, specific hypotheses being tested.**

**Statement:** The objectives of the present study were to i) to elucidate the effect of dietary chitosan on growth, biochemical composition and hematological parameter ii) to assess the effect of dietary chitosan on gut microbiota status and digestive enzymes of juvenile *B. gonionotus* and on pathogenic bacteria of fishes and iii) to analyze the effect of dietary chitosan on the histology of intestine, liver and kidney of juvenile *B. gonionotus*.

**Item 14. Ethical statement**

**Provide the name of the ethical review committee or equivalent that has approved the use of animals in this study and any relevant licence or protocol numbers (if applicable). If ethical approval was not sought or granted, provide a justification.**

**Statement:** The use of animals was kept to an absolute minimum required to achieve statistical significance for validation purposes. All procedures were conducted in accordance with the United Kingdom Animal (Scientific Procedures) Act 1986, approved by Ethical Review Committee (ERC) of the Institute of Biotechnology and Genetic Engineering (IBGE), Bangabandhu Sheikh Mujibur Rahman Agricultural University (BSMRAU), Gazipur-1706, Bangladesh and conducted under the authority of the project Licence BSMRAU/IBGE/003.

**Item 15. Housing and husbandry**

**Provide details of housing and husbandry conditions, including any environmental enrichment.**

**Statement:** The fish were housed in circular plastic tanks (500L) with continuous aeration. Water change was done every 3 days interval and uneaten feeds were collected. Water quality parameters such as, pH, dissolve oxygen, and temperature were routinely measured to maintain the health of fish.

**Item 16. Animal care and monitoring**

**16a. Describe any interventions or steps taken in the experimental protocols to reduce pain, suffering, and distress.**

**Statement:** Prior blood collection, the experimental fish were anesthetized by using 0.05 mL clove oil per 500 mL of water to reduce pain, suffering, and distress.

**16b. Report any expected or unexpected adverse events.**

**Statement:** No adverse events were noticed during the study period.

**16c. Describe the humane endpoints established for the study, the signs that were monitored, and the frequency of monitoring. If the study did not set humane endpoints, state this.**

**Statement:** The research team monitored the experimental fish twice daily. Health was monitored by food intake. Water temperature, dissolved oxygen (DO), and pH of water in each replication under each treatment were recorded every day.

**Item 17. Interpretation/scientific implications**

**17a. Interpret the results, taking into account the study objectives and hypotheses, current theory, and other relevant studies in the literature.**

**Statement:** In summary, the dietary chitosan treated formulated feeds resulted in promotion of growth and nutrient composition in the muscles, positive changes in gut microbial status, the digestive enzyme activities, haematological parameter and the intestinal, liver and kidney morphology of juvenile *B. gonionotus* fishes. However, these dietary chitosan had no detectable effects on mineral contents in the muscles of *B. gonionotus*. Our findings suggest that the dietary chitosan at the dose of 1 g kg^-1^ feed could be supplemented to a practical diet to enhance growth and development of cultured *B. gonionotus*.

**17b. Comment on the study limitations, including potential sources of bias, limitations of the animal model, and imprecision associated with the results.**

**Statement:** Although in this study we collected experimental fish samples from only one source. We did not analyse the immune gene expression of *B. gonionotus*.

**Item 18. Generalisability/translation**

**Comment on whether, and how, the findings of this study are likely to generalise to other species or experimental conditions, including any relevance to human biology (where appropriate).**

**Statement:** Our study demonstrated the effects of dietary chitosan on growth, biochemical composition, hematological parameter and morphology of internal organs of *B. gonionotus*. Further studies should be focused on the elucidation of underlying mechanisms of the beneficial effects of the dietary chitosan on the fishes.

**Item 19. Protocol registration**

**Provide a statement indicating whether a protocol (including the research question, key design features, and analysis plan) was prepared before the study, and if and where this protocol was registered.**

**Statement:** To maximize the objectivity of the presented research, we preregistered this study with its 2 hypotheses at the Open Science Framework (OSF) ([www.doi.org/10.17605/OSF.IO/4YABR](http://www.doi.org/10.17605/OSF.IO/4YABR)).

**Item 20. Data access**

**Provide a statement describing if and where study data are available.**

**Statement:** Not applicable in this research.

**Item 21. Declaration of interests**

**21a. Declare any potential conflicts of interest, including financial and nonfinancial. If none exist, this should be stated.**

**Statement:** The authors have declared that no competing interests exist.

**21b. List all funding sources (including grant identifier) and the role of the funder(s) in the design, analysis, and reporting of the study.**

**Statement:** This research was mainly funded by a grand awarded to Mohammad Abdus Salam, Dept. of Genetics and Fish Breeding by Research Management Wing (RMW), Bangabandhu Sheikh Mujibur Rahman Agricultural University (BSMRAU), Bangladesh (Project ID No.: Ref. No. D(R)/ (RMC-PE) 2017-2020(38).The funders had no role in study design, data collection and analysis, decision to publish, or preparation of the manuscript.
