# Supplementary material for: Dietary chitosan promotes the growth, biochemical composition, gut microbiota, hematological parameters and internal organ morphology of juvenile Barbonymus gonionotus
Source: PLoS One. 2021 Nov 18;16(11):e0260192. doi: 10.1371/journal.pone.0260192 (PMC8601453; doi:10.1371/journal.pone.0260192)
Supplement: S1 Table — (DOCX) [file pone.0260192.s002.docx]

**S2 Table. Exact feed consumed by fish and nutrient composition of that feed.**

|  | **Treatment** | | | |
| --- | --- | --- | --- | --- |
|  | **Control** | **T1** | **T2** | **T3** |
| Food intake (%) | 80 | 87 | 85 | 83 |
| **Nutrient composition** | | | | |
| Protein (%) | 25.90 | 33.41 | 30.79 | 28.07 |
| Lipid (%) | 7.00 | 9.03 | 8.32 | 7.58 |
| Carbohydrate (%)/ NFE (%) | 22.40 | 28.89 | 26.63 | 24.27 |
| Ash (%) | 9.86 | 12.64 | 11.65 | 10.62 |
| Crude fiber (%) | 4.22 | 5.52 | 4.99 | 4.55 |

Here, NFE = nitrogen free extract
